# Phuphan chicken breeds: classification as varieties or distinct breeds with three derivative groups using microsatellite genotyping

**DOI:** 10.5713/ab.24.0579

**Published:** 2025-05-19

**Authors:** Ekerette Ekerette, Nivit Tanglertpaibul, Trifan Budi, Wisut Auekingpetch, Chien Phuoc Tran Nguyen, Worapong Singchat, Wongsathit Wongloet, Nichakorn Kumnan, Piangjai Chalermwong, Anh Huynh Luu, Thitipong Panthum, Aingorn Chaiyes, Kanithaporn Vangnai, Chotika Yokthongwattana, Chomdao Sinthuvanich, Narongrit Muangmai, Prateep Duengkae, Kornsorn Srikulnath

**Affiliations:** 1 Animal Genomics and Bioresource Research Unit (AGB Research Unit), Faculty of Science, Kasetsart University, Bangkok, Thailand; 2 Animal Genetics and Genomic Unit, Department of Genetics and Biotechnology, University of Calabar, Calabar, Nigeria; 3 Interdisciplinary Graduate Program in Bioscience, Faculty of Science, Kasetsart University, Bangkok, Thailand; 4 Phuphan Royal Development Study Centre, Office of the Royal Development Projects Board (ORDPB), Sakhon Nakhon, Thailand; 5 Department of Livestock Development, Ministry of Agriculture and Cooperative, Bangkok, Thailand; 6 Special Research Unit for Wildlife Genomics (SRUWG), Department of Forest Biology, Faculty of Forestry, Kasetsart University, Bangkok, Thailand; 7 School of Agriculture and Cooperatives, Sukhothai Thammathirat Open University, Nonthaburi, Thailand; 8 Department of Food Science and Technology, Faculty of Agro-Industry, Kasetsart University, Bangkok, Thailand; 9 Department of Biochemistry, Faculty of Science, Kasetsart University, Bangkok, Thailand; 10 Department of Fishery Biology, Faculty of Fisheries, Kasetsart University, Bangkok, Thailand; 11 Laboratory of Animal Cytogenetics and Comparative Genomics (ACCG), Department of Genetics, Faculty of Science, Kasetsart University, Bangkok, Thailand; 12 Biodiversity Center Kasetsart University (BDCKU), Bangkok, Thailand

**Keywords:** Black-boned Chicken, Breed, Genetic Cluster, Indigenous Chicken, Microsatellite, Variety

## Abstract

**Objective:**

Indigenous and local breeds, such as Phuphan chickens, are vital due to their adaptability and nutritional value. However, the precise origin, historical records, and genetic diversity of Phuphan chickens remain unclear. This study aimed to evaluate origin and genetic diversity of four Phuphan chicken groups from the Phuphan Royal Development Study Centre.

**Methods:**

This study assesses four groups of Phuphan chicken: Phuphan black 1 (SK-B1), Phuphan black 2 (KU-BM/F), Phuphan white (KU-WM/F), and Phuphan color (KU-VM/F) using 28 microsatellite markers and comparing them with those of other Thai chicken breeds within “The Siam Chicken Bioresource Project” database.

**Results:**

The results highlighted significant genetic diversity among these groups (mean expected heterozygosity [*H*_e_] = 0.623±0.014; Allelic richness [*AR*] = 4.594±0.124), indicating effective management through the breeding program of the Phuphan Royal Development Study Centre. Population structure analyses revealed distinct gene pools, emphasizing the genetic uniqueness of SK-B1 relative to the other three groups. Bayesian inference validated historical genetic exchanges, primarily among KU-BM/F, KU-WM/F, and KU-VM/F, with limited exchanges involving SK-B1. This suggests that the Phuphan chicken groups share a common lineage, primarily distinguished by variations in plumage color, resulting from residual selection processes. Microsatellite markers pinpointed the loci LEI0234, MCW206, MCW0016, MCW0222, MCW0098, MCW0165, and ADL0278 as potentially subject to directional selection and associated with plumage color variation among the Phuphan chicken groups. Comparative evaluations with other Thai indigenous local chickens and red junglefowl revealed a closer affinity of SK-B1 to existing Thai chicken breeds, suggesting it may represent a variant of these breeds. Alternatively, KU-BM/F, KU-WM/F, and KU-VM/F, which exhibited comparable external characteristics, may constitute a novel breed of Phuphan chicken.

**Conclusion:**

The findings may enhance understanding on genetic architecture of Phuphan chicken groups and contribute to Thailand’s economic growth while preserving the genetic diversity of the indigenous chickens.

## INTRODUCTION

As the global population nears 9.8 billion by 2050 [1], the demand for poultry products is increasing. Fast-growing commercial chicken breeds have been prioritized, diminishing the role of indigenous and local chicken breeds in sustainable poultry production. Poultry growth slowed, similar to that of indigenous and local chicken breeds, in 2022 (0.73%) owing to changing climatic conditions [2]. However, indigenous and local chicken breeds are recognized for their genetic advantages and better resilience [3]. Indigenous and local chicken breeds harbor many variations, which are often shaped by their unique environments. Their adaptability and low maintenance requirements are considered crucial for smallholder farming in developing countries such as Thailand [4]. A well-known indigenous and local chicken breed in Thailand is the black-bone chicken, which is recognized for its black skin, meat, and bones. These characteristics are attributed to melanism and fibromelanosis [5]. The blackbone variety offers low fat and cholesterol, high protein and collagen levels, and higher levels of carnosine and anserine than that of the commercial breeds [5]. Based on historical records, most blackbone chickens in Thailand were brought by Chinese immigrants several decades ago. Breeds such as Chee Fah and Fah Luang in Chiang Rai Province have developed and adapted to the local environment, becoming local breeds that are deeply rooted in sociocultural communities [6]. Similar cases of the development of resilient indigenous and local chicken breeds with economic value have been reported globally. Examples include the Ri chicken breed in Vietnam [7], the Sakini chicken breed in Nepal [8], the Ninghai chicken breed in China [9], and the Fulani ecotype in Nigeria [10]. Remarkably, novel characteristics are occasionally observed in individual chickens within a flock. If these traits are economically valuable, developers will select and work on them, eventually developing a new variety of local breeds or even new breeds.

Phuphan chicken, a black-boned breed, was developed by the Phuphan Royal Development Study Centre and Thailand’s Department of Livestock. Although the precise origin and historical records of the Phuphan chicken remain unclear, five local black-bone chickens from Sakon Nakhon Province are the original great-grandparents of this flock. However, through domestication and maintenance processes, a pure line of Phuphan chickens has been developed since 2002 [11], which was distinguished by a uniform black plumage. Male Phuphan blackbone chickens are noted to weigh 2.5–3.0 kg, whereas females weigh 1.5–2.0 kg. Egg-laying maturity was observed at 21–22 weeks [11]. In Asian markets, high values are placed on poultry’s black skin and meat quality. High melanin content acts as a protective agent against environmental stress and a natural antioxidant and is used in traditional Chinese medicine [12]. This breed provides food security and rural development to local communities. Notably, during the maintenance of the pure Phuphan chicken breed, new plumage color traits occurred, likely through mutation, although the meat and skin remained black. The new plumage color traits are managed separately to retain the pure varieties of each group. These new Phuphan chicken varieties are being promoted to establish other pure lines with potential ornamental uses. Ongoing breeding efforts have developed: (i) Phuphan white, with white feathers; (ii) Phuphan golden feathers, with a golden feather pattern and black tail; and (iii) Phuphan mixed color, featuring gold and white feathers with a black tail. The promotion of Phuphan chickens could boost the local bioeconomy and attract consumer preference [6,13]. However, identifying and classifying local chickens is crucial to prove breed differences. Evidence is required to demonstrate the separation between new and existing breeds before promotion, registration, and commercial development. Relying solely on morphological traits, such as meat quality and ornamental appearance, may be insufficient to differentiate between breeds or varieties because of narrow distinctions. DNA fingerprinting was used as a backup approach to confirm conclusive evidence. Given their importance in sustainable agriculture, refining methods for evaluating and characterizing Thai indigenous and local chickens are essential for effective breeding programs.

This study addressed the Phuphan chicken breed and variety levels using comprehensive genomic studies and examined the genetic diversity of 28 microsatellite markers. The results were compared with data from an extensive gene pool library obtained from “The Siam Chicken Bioresource Project, SCBP” (https://www.sci.ku.ac.th/scbp/ and Dryad dataset: https://datadryad.org/stash/share/x2qlPmboMgCROXO8, accessed on January 15, 2024) to examine the genetic structures of Phuphan chicken [14,15]. Genetic introgression and gene pool sharing with other Thai indigenous chickens may have occurred, questioning the genetic purity of Phuphan chickens. The following hypotheses were tested: (1) distinct gene pools of Phuphan chickens, which prove that they are different breeds from other indigenous and local chicken breeds in Thailand, are revealed through genetic testing; (2) a high level of inbreeding is observed in Phuphan chickens, as all individuals were historically developed from five original individuals; and (3) different plumages of Phuphan chickens might indicate the differentiation of several varieties. With the explicit goal of preserving the genetic diversity in genetic stocks, these chicken breeds may serve as vital repositories of invaluable genetic information, contributing to rural development.

## MATERIALS AND METHODS

### Specimen collection and DNA extraction

**Figure 1:**
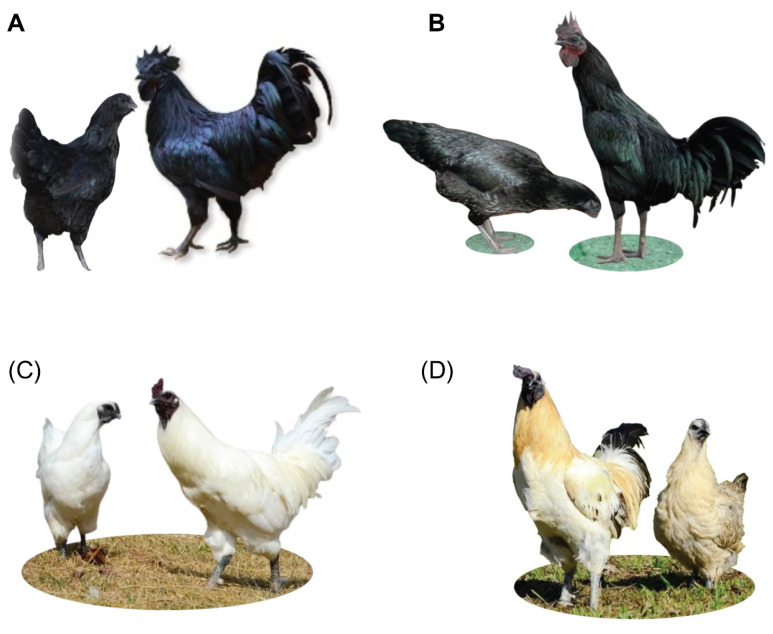
Phuphan chickens varieties used in this study. (A) Phuphan black 1 (SK-B1), (B) Phuphan black 2 (KU-BM/F), (C) Phuphan white (KU-WM/F), and (D) Phuphan color (KU-VM/F).

Blood specimens were collected from 90 Phuphan chickens divided into four groups (Phuphan black 1 [SK-B1; n = 30], Phuphan black 2 [KU-BM/F; n = 20], Phuphan white [KU-WM/F; n = 20], and Phuphan color [KU-VM/F; n = 20; Figure 1; Supplement 1]). Specimen collection, DNA extraction, and DNA qualification and quantification were performed as described previously [14].

### Microsatellite genotyping and data analysis

Twenty-eight microsatellite primer sets based on the recommendations of the Food and Agriculture Organization (FAO) were used for genotyping to assess the biodiversity of the chicken populations [16]. The 5′-end of each forward primer was labeled with either 6-fluorescein amidite (6-FAM) or hexachlorofluorescein (HEX; Macrogen, Seoul, Korea). Microsatellite PCR amplification was performed in triplicate for each sample, following the method described previously by [14], to ensure accurate results and minimize the chance of false allele amplification. Genetic diversity was assessed by calculating the observed and expected heterozygosity (*H*_o_ and *H*_e_), allelic richness (*AR*), polymorphic information content (*PIC*), number of alleles per group (*N*_a_), *F*-statistics (*F*_IS_ and *F*_ST_), relatedness (*r*), and pairwise Nei’s genetic distance in GenAlex [17]. Population structure analyses, such as analysis of molecular variance (AMOVA), principal coordinate analysis (PCoA), discriminant analysis of principal components (DAPC), and STRUCTURE analysis, were performed following [14]. The Wilcoxon signed-rank test for detecting recent population bottlenecks was performed using a two-phase mutation model (TPM) and a stepwise mutation model (SMM) to assess the probability of excess heterozygosity due to small sample sizes. The allelic range was obtained for each locus using Arlequin version 3.5 [18], which was used to calculate the relative long-term bottleneck events based on the *M* ratio. A selective sweep analysis was performed, in which the *H*_e_ and *F*_IS_ values of the chicken groups were plotted for each microsatellite locus (28 loci in total), as described previously [14]. High *F*_IS_ and low *H*_e_ values reflect sweeping or purifying/negative selection, whereas low *F*_IS_ and high *H*_e_ values indicate neutral or balanced selection [19]. Microsatellite locus neutrality was assessed using the Bayesian regression approach in BAYESCAN [20], which calculates the Bayes factor to estimate the probability of locus selection. This factor represents the ratio of the posterior probabilities of the two models, selection and neutral, based on data.

To examine the occurrence of genetic exchange between Phuphan chicken groups based on the microsatellite genotyping dataset, Bayesian interference analysis implemented in BayesAss version 3.0.5 [21], which is commonly used to determine recent migration rates between populations, was performed. Markov Chain Monte Carlo (MCMC) analysis was conducted for 10 million generations after a burn of 1 million generations and sampled every 100 generations. The mixing parameters associated with migration rates (*m*), allele frequencies (*a*), and inbreeding coefficients (*f)* were optimized to satisfy 20%–60% posterior distribution acceptance rates according to the recommended guidelines [21]. Similarly, to assess the historical genetic exchange between Phuphan chicken groups, migration rates between the groups and their effective population sizes were estimated by Bayesian analysis using MIGRATE-N version 4.4.3 [22]. Uniform prior distributions were used for the basic microsatellite model, and 5,000 steps were recorded every 100 generations using the MCMC procedure. The first 100,000 generations are discarded as burn-ins. Estimates were calculated for the mutation-scaled immigration rate (*M*) and mutation-scaled population size (Θ). The number of individuals entering populations (*N*_m_) was calculated, and the presence of gene flow between populations in the past was determined using the formula *N*_mi–>j_ = Θ_j_*M_i–>j_/4, where *N*_mi–>j_ represents the effective number of immigrants or gene flow rate from population *i* to population *j* per generation. Circos version 0.69–8 was used to visualize genetic connectivity among populations [23]. The genotypic data generated in this study are stored in the Dryad Digital Repository dataset (https://doi.org/10.5061/dryad.hhmgqnkm0; accessed on 1 August 2024).

### Investigation of the genetic origins of Phuphan chicken groups

The genetic origins of the Phuphan chicken groups were investigated using microsatellite genotyping data of chickens available under the SCBP (https://www.sci.ku.ac.th/scbp/; https://doi.org/10.5061/dryad.hhmgqnkm0, accessed on 1 August 2024), including red junglefowl and indigenous and local chicken breeds in Thailand. All indigenous and local chicken populations were treated as separate populations. Pairwise genetic distances between populations and clustering analyses based on PCoA, DAPC, and STRUCTURE were performed as previously described [14].

## RESULTS

### Genetic diversity of the Phuphan chicken group based on microsatellite genotyping data

A total of 548 alleles were observed in the four groups of Phuphan chicken, with the mean number of alleles per locus as 4.893±0.204 (Table 1). All allelic frequencies showed a significant departure from Hardy–Weinberg equilibrium, with multiple lines of evidence for linkage disequilibrium. Null alleles were frequently found at the MCW0016, MCW0014, MCW0034, MCW0037, MCW0216, MCW0248, MCW0165, MCW0104, MCW0069, LEI0094, LEI0192, and ADL0278 loci; nevertheless, all markers were similarly treated. The *F* values for all Phuphan chicken groups were negative, except for the SK-B1 group (Table 1 and Supplement 2). The *PIC* of all Phuphan chicken groups ranged from 0.556 to 0.592, whereas the Shannon’s Information Index (*I*) was from 1.167 to 1.267 with an average of 1.201±0.039. The mean *H*_o_ and *H*_e_ values were 0.639±0.022 and 0.623±0.014, respectively (Table 1). The mean effective number of alleles in four Phuphan chicken groups was 3.112±0.125. The mean *AR* values for the four Phuphan groups was 4.594±0.124. The standard genetic diversity indices are summarized in Table 1. Welch’s t-test revealed significant differences (p<0.05) between *H*_o_ and *H*_e_ in the SK-B1 and KU-VM/F groups, whereas the KU-BM/F and KU-WM/F groups were not significantly different. The pairwise comparison of *H*_o_ and *H*_e_ revealed a significant difference in *H*_o_ between SK-B1 and KU-BM/F, SK-B1 and KU-WM/F, and SK-B1 and KU-VM/F, while *H*_e_ did not show any statistical difference between groups. The average *F*_IS_ values for SK-B1, KU-BM/F, KU-WM/F, and KU-VM/F were −0.036, −0.016, −0.026, and −0.063, respectively. The mean *r* values evaluated for the four Phuphan chicken groups were −0.022, −0.026, −0.026, and −0.025 for SK-B1, KU-BM/F, KU-WM/F, and KU-VM/F, respectively. However, the distributions of *F*_IS_ and *r* among the chicken groups were not significantly different. The *F*_ST_ value was significant (p<0.05) for all group combinations after 110 permutations (Supplement 3). AMOVA based on 28 microsatellite loci revealed 14% variation among groups and 5% variation among individuals within groups (Supplement 4). The Nei genetic distance revealed the highest value of 0.667 between SK-B1 and KU-BM/F and the lowest distance of 0.088 between KU-WM/F and KU-VM/F (Supplement 5).

The PCoA and DAPC results classified the Phuphan chickens into two clusters. SK-B1 cells formed a distinct cluster, whereas KU-BM/F, KU-WM/F, and KU-VM/F were intermixed within another cluster (Supplements 6, 7). Different gene pool patterns between populations were observed using model-based Bayesian algorithms implemented in STRUCTURE with increased *K*-values (*K* = 1 to *K* = 25; Supplement 8). The highest posterior probability value was found at *K* = 2 based on Evanno’s Δ*K*, while the mean In P(*K*) had the highest probability value at *K =* 4. At *K* = 2, SK-B1 showed different gene pool patterns, whereas KU-BM/F, KU-WM/F, and KU-VM/F showed similar gene pool patterns. At *K* = 4, all four groups showed distinct gene pool patterns with some evidence of admixture in the KU-BM/F, KU-WM/F, and KU-VM/F populations. Similarly, at the highest *K*-value (*K* = 25), all four groups showed distinct gene pool patterns, with evidence of an admixture in the KU-BM/F, KU-WM/F, and KU-VM/F populations (Supplement 8). The genetic selective sweep plot revealed higher *H*_e_ values than *F*_IS_ in the 28 microsatellite loci and the four Phuphan chicken groups, indicating neutral or balanced selection (Supplement 9). Loci LEI0234, MCW206, MCW0016, MCW0222, MCW0098, MCW0165, and ADL0278 were identified using the BAYESCAN approach as having an infinite probability of being under directional selection (Supplement 10). The Wilcoxon signed-rank test ranged from 0.082 to 0.323 and 0.505 to 0.373 for TPM and SMM, respectively, with a normal L-shaped mode shift, indicating the absence of a recent bottleneck in Phuphan chickens. The *M* ratios were lower than the 0.68 threshold by Garza and Williamson [24], suggesting a rapid population decline in these chicken groups through historical times (Table 1 and Supplement 2). The current gene flow, indicated by the migration rate, ranged from 0.681 to 0.971 within the group and 0.01 to 0.059 between the groups. MIGRATE-N analysis revealed mode values of 0.001 for KU-BM/F, KU-WM/F, and KU-VM/F, whereas the SK-B1 group had a mode value of 0.098. The asymmetric migration rate ranged from 4.333 to 989.667, with the highest values observed in KU-VM/F and KU-WM/F. The effective number of immigrants (*N_m_*) ranged from 0.001 to 0.220, with the highest value observed between SK-B1 and KU-BM/F (0.220), indicating the possibility of high gene flow between these two chicken groups. All the calculation results are available from the Dryad Digital Repository Dataset (https://doi.org/10.5061/dryad.hhmgqnkm0, accessed on 1 August 2024).

### Genetic differences among Phuphan chicken groups, red junglefowl, and other indigenous and local chicken breeds in Thailand

**Figure 2:**
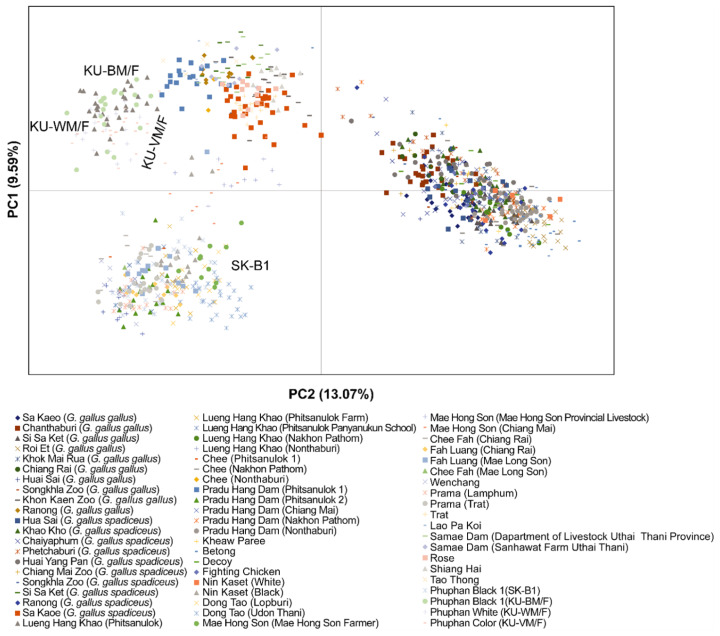
Principal coordinate analysis (PCoA) of four Phuphan chicken varieties, red junglefowl, and domestic chicken breeds based on 28 microsatellite loci. Different populations/breeds are represented by different colors.

**Figure 3:**
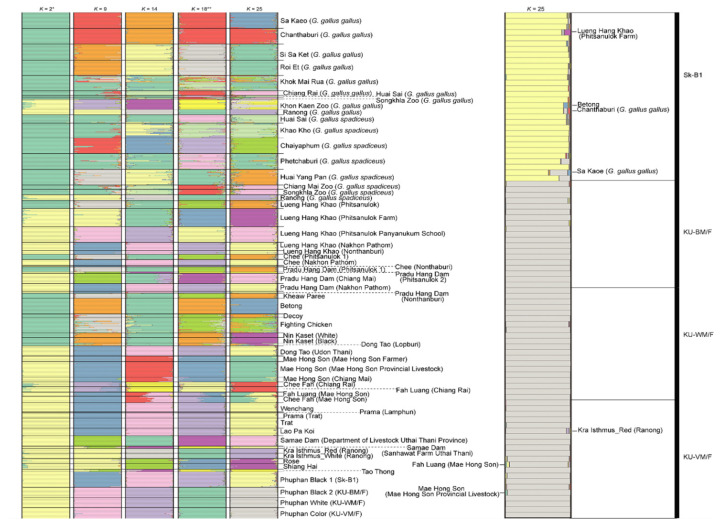
Population structure of four Phuphan chicken varieties with red junglefowl and domestic breeds in Thailand. The x-axis represents the proportion of membership (posterior probability) in each genetic cluster, while each horizontal bar on the y-axis represents an individual. All individuals from the four varieties are superimposed on the plot. Black vertical lines indicate the boundaries. The highest posterior probability, denoted by * was determined based on Evanno’s Δ*K*, and the highest ln P(*K*) is represented by **.

AMOVA analysis of Phuphan chickens with those from other indigenous and local chicken breeds and red junglefowl in Thailand revealed higher variation within the group (75.44%) than among groups (24.56%). In PCoA, the Phuphan chickens were grouped into clusters similar to those of other indigenous chicken breeds (Figure 2). SK-B1 was clustered with Lueng Hang Khao (Nakhon Pathom), Pradu Hang Dam (Phitsanulok 2), Chee Fah (Chiang Rai), Prama (Trat) Chee Fah (Mae Hong Son), and Nin Kaset (Black). By contrast, KU-BM/F, KU-WM/F, and KU-VM/F groups were grouped with Lueng Hang Khao (Phitsanulok). The DAPC grouped the KU-BM/F, KU-WM/F, and KU-VM/F groups independently under one cluster, except for SK-B1 individuals, which appeared to be intermixed with other indigenous and local chicken breeds. Using the microsatellite genotyping dataset from our previous investigations on indigenous and local chickens, along with the red junglefowl in Thailand (accessible at SCBP, https://www.sci.ku.ac.th/scbp/), a comparative analysis of the Phuphan chicken gene pool using STRUCTURE analysis was conducted, which revealed different gene pool patterns with increased in *K*-values (*K* = 1 to *K* = 25). The highest posterior probability based on Evanno’s Δ*K* was at *K* = 2, while the mean In P(*K*) had the highest peak at *K* = 18 (Figure 3 and Supplement 11). At *K* = 2, the four groups of Phuphan chickens shared similar gene pool patterns with many indigenous and local chicken breeds in Thailand, including Tao Tong, Lao Pa Koi, Trat, Wenchang, Prama, Chee Fah, Fah Luang, Mae Hong Son, Dong Tao, Pradu Hang Dam (Phitsanulok 1), Pradu Hang Dam (Phitsanulok 2), Pradu Hang Dam (Chiang Mai), Lueng Hang Khao (Phitsanulok Panyanukun School), and Lueng Hang Khao (Nakhon Pathom). At *K*= 18, SK-B1 shared a gene pool pattern similar to that of indigenous chickens such as Lao Pa Koi, Trat, Prama, Wechang, Dong Tao, Lueng Hang Khao (Nakhon Pathom), Chee, Pradu Hang Dam (Nonthaburi), Pradu Hang Dam (Nakhon Pathom), and red junglefowl derived from Chaiyaphum (*Gallus gallus spadiceus*). SK-B1 also shared a partial gene pool pattern with Chee Fah and Fah Luang strains. KU-BM/F, KU-WM/F, and KU-VM/F shared similar gene pools with Kra Isthmus_Red, Kra Isthmus_White, and red junglefowl derived from Huai Yang Pan (*G. gallus spadiceus*) and Ranong (*G. gallus spadiceus* and *G. gallus gallus*). Within higher *K*-value (*K* = 25), SK-B1 was observed to share similar gene pool pattern with Samae Dam (Sanhawat Farm Uthai Thani), Lao Pa Koi, Trat, Prama, Wechang, Dong Tao, Pradu Hang Dam (Nakhon Pathom), Pradu Hang Dam (Nonthaburi), Lueng Hang Khao (Nakhon Pathom), Lueng Hang Khao (Nonthaburi), Chee (Nakhon Pathom), and Chee (Nothanburi). SK-B1 shares partial gene pool patterns with the Chee Fah and Fah Luang chickens. KU-BM/F, KU-WM/F, and KU-VM/F showed distinct gene pool patterns that were not shared by other chicken breeds or red junglefowl (Figure 3). The detailed results of all the calculations are available in the Dryad Digital Repository (https://doi.org/10.5061/dryad.hhmgqnkm0; accessed on 1 August 2024).

## DISCUSSION

The genetic makeup of Phuphan chicken varieties has been investigated to elucidate their origin and assess potential introgression from other Thai breeds since their 2002 establishment as Chinese blackbone chickens in Sakon Nakhon Province. Ongoing efforts by the Phuphan Royal Development Study Centre and Thai Department of Livestock have led to the development of four distinct groups (Phuphan Royal Development Study Centre, Personal Communication). Understanding genetic diversity is essential for enhancing the utilization of Phuphan chickens as a genetic resource. Genetic diversity, measured by allele numbers, frequencies, and heterozygosity, is influenced by population size, mating patterns, and natural selection, and provides resilience against environmental changes, diseases, and other challenges. Understanding the genetic diversity in indigenous breeds can bolster utilization efforts and inform strategic breeding programs for future use [3,8]. In this study, all groups of Phuphan chickens exhibited high genetic diversity (mean *H*_e_ = 0.623±0.014; *AR* = 4.594±0.124), suggesting effective management of Phuphan chicken varieties by the Phuphan Royal Development Study Centre since their development in 2002. This was further confirmed by the low *F*_IS_ and *r* values, with no evidence of bottleneck events during the breeding process, thus validating the effective management strategies for Phuphan chickens. Low *AR* values are often observed in captive domestic chickens or in new varieties of domestic chickens [25]. Significant genetic differentiation (average *F*_ST_ = 0.130) was observed among the groups. High clustering success rates (>90%) were achieved by genotyping at least 15 individuals per group/population, using a minimum of 15 highly informative microsatellite loci. Genetic assignment accuracy approaches 100% if *F*_ST_ values exceed 0.10 with more than 20 loci, ensuring the accuracy of subsequent clustering analyses [26].

### Emergence of new traits in Phuphan chickens through remnant selection processes

Analysis of the population structure revealed distinct gene pool patterns among the four Phuphan chicken groups, dividing them into two major clusters across varying *K* values (*K =* 2 to *K =* 25). Specifically, SK-B1 exhibited a unique gene pool pattern that differed notably from that of KU-BM/F, KU-WM/F, and KU-VM/F, which shared a similar genetic makeup at *K* = 2 and *K* = 4 in the STRUCTURE analysis, consistent with both the PCoA and DAPC results. This suggests that SK-B1 has less genetic similarity to the other Phuphan groups, as confirmed by the higher *F*_ST_ and *D* values between SK-B1 and the other three groups. By contrast, genetic admixture was observed in KU-BM/F, KU-WM/F, and KU-VM/F, indicating genetic exchange among these groups, as confirmed by PCoA and DAPC. Bayesian inference analysis indicated a historically high genetic exchange between KU-BM/F, KU-WM/F, and KU-VM/F but a very low exchange with SK-B1. This suggests that the different Phuphan chicken groups, primarily those based on external morphological characteristics, share a common ancestral lineage. Selective management has fostered the development of distinct groups currently bred at the Phuphan Royal Development Study Centre. Historical information indicates that the original Phuphan chicken, likely SK-B1, is black. A new white trait occurred accidentally in the KU-BM/F group, leading to selective management and propagation within the Phuphan Royal Development Study Centre. A similar case was observed in the color group (KU-VM/F) of Phuphan chickens (Phuphan Royal Development Study Centre, Personal Communication). This suggests that SK-B1 likely developed as a foundational Phuphan chicken group through continuous selective management of Phuphan black-bone chickens in Sakon Nakhon. Other groups exhibiting variations in external morphology, plumage color, or both can be classified as groups originating during the early phase of the Phuphan Royal Development Study Centre’s breeding program. Different groups of Phuphan chickens were maintained separately to retain the pure lines of each group. The present findings are consistent with those of previous studies that used microsatellite markers to distinguish morphologically similar indigenous chickens [14,27].

It is likely that certain microsatellite loci may be linked to adaptive genes that influence plumage color [28–30]. This linkage may have contributed to the development of the KU-BM/F, KU-WM/F, and KU-VM/F groups from SK-B1, which are distinguished by their distinct plumage colors. Notably, BAYESCAN analysis identified LEI0234, MCW206, MCW0016, MCW0222, MCW0098, MCW0165, and ADL0278 as loci undergoing directional selection in the Phuphan chicken groups. These loci may correspond to regions harboring these adaptive genes, potentially influencing the differentiation of Phuphan chickens into distinct phenotypic groups. Several studies have identified candidate genes and genomic regions responsible for plumage color in chickens, such as *EGR1, MLPH, RAB17, SOX5, GRM5* and other genes within chromosomes 1, 3, 4, 8, 12, 21, and 24 [28–30], whereas the loci under selection were located on chromosomes 2, 3, 4, 8, and 23 [16]. This suggests that potential signatures of hitchhiking selection affect microsatellite loci. Selection of a functional gene can strongly influence the allelic frequency at nearby tightly linked loci, even if these microsatellite loci themselves are selectively neutral [31]. Although specific adaptive genes or mutations were not directly assessed, this microsatellite study highlights the potential for future research on the genomic regions responsible for phenotypic variation, particularly plumage color, in Phuphan chickens. High-throughput genomic methods such as SNP analysis, ddRAD sequencing, and whole-genome sequencing, complemented by robust data analysis, offer promising avenues for elucidating genomic changes [3,32–35].

### Two explanations of genetic groups in Phuphan chickens at breed and variety levels

Results from PCoA, DAPC, and STRUCTURE analyses using datasets from Phuphan chickens and SCBP indicated that SK-B1 clustered with other Thai indigenous breeds such as Lueng Hang Khao (Nakhon Pathom), Pradu Hang Dam (Phitsanulok 2), Chee Fah (Chiang Rai), Prama (Trat), Chee Fah (Mae Hong Son), and Nin Kaset (Black). In contrast, KU-BM/F, KU-WM/F, and KU-VM/F cells did not exhibit similar clusters. This suggests that SK-B1 is more genetically related to other Thai indigenous and local chicken breeds and red junglefowl than to KU-BM/F, KU-WM/F, and KU-VM/F. This finding aligns with records indicating the use of Chinese black-boned chickens as the founding stock, with subsequent groups developing from SK-B1 [11]. Previous SCBP studies have shown that microsatellite markers can reliably distinguish Thai chicken breeds, which vary in traits such as weight, plumage, body shape, meat quality, and fecundity [14,27, 36,37]. Although useful for genetic diversity insights, microsatellites lack the resolution required to identify specific genes linked to phenotypic variations [38]. The status of the two divergent groups of Phuphan chickens may be explained by two hypotheses. Phuphan chickens in all groups (SK-B1, KU-BM/F, KU-WM/F, and KU-VM/F) were classified as Phuphan chicken breeds with unique morphological characteristics compared to other Thai indigenous and local chicken breeds. SK-B1 may differ from KU-BM/F, KU-WM/F, and KU-VM/F at various levels owing to its utilization and domestication processes. SK-B1 is primarily consumed locally, as it grouped with Thai indigenous and local chicken breeds used for consumptions, such as Mae Hong Son, Chee Fah, and Fah Luang chickens (Figures 2, 3). By contrast, KU-WM/F and KU-VM/F are used for ornamental purposes, as they are genetically closer to Thai indigenous and local chicken breeds designated for ornamental purposes such as Lueng Hang Khao derived from Phitsanulok Panyanukun School [27] as indicated by PCoA and STRUCTURE analyses. This distinction may result in different strain levels based on plumage color. Alternatively, SK-B1 of Phuphan chickens showed high genotypic similarity with other Thai indigenous breeds, suggesting that it may not be classified as a new breed, but rather as a variation within known chicken breeds. However, KU-BM/F, KU-WM/F, and KU-VM/F, which retain the external morphology of Phuphan chickens and have undergone gradual genetic separation through selective management, might represent a new Phuphan chicken breed. These varieties, KU-BM/F, KU-WM/F, and KU-VM/F, were distinguished by their plumage color. Further genomic studies are required to examine the functional genes of each variety or strain. High-throughput genotypic methods are recommended to assess the genes responsible for phenotypic differences among the four Phuphan chicken varieties [39]. Preserving the genetic diversity of Phuphan chickens is crucial for enhancing the productivity, livelihood, and profitability of farmers. These chickens, which are well suited for various regions in Thailand, are noted for higher egg production, lower feed requirements, and faster maturation than other black-bone breeds [5,6]. Understanding the genetic architecture through comprehensive data analysis is essential for future breeding programs and can significantly contribute to Thailand’s economic growth. As part of the SCBP’s initiative at Kasetsart University to characterize indigenous and local chicken breeds, collaboration with local communities and stakeholders is integral to promoting Phuphan chickens as a valuable genetic resource for Thailand’s breeding efforts.

## CONCLUSION

The preservation and harnessing of the genetic diversity of Phuphan chickens can bolster sustainable food production and genetic conservation efforts in poultry breeding programs. Preliminary genetic studies must be conducted to gain insights into the genetic architecture of Phuphan chickens. Substantial genetic diversity and differentiation among the SK-B1, KU-BM/F, KU-WM/F, and KU-VM/F groups were observed in this study. Population structure analyses delineated distinct gene pools, with SK-B1 identified as genetically unique compared to other groups. This suggests that the four Phuphan chicken groups exhibited unique morphologies, with SK-B1 differing at the variety level due to utilization and domestication. Alternatively, comparative evaluations with other Thai indigenous chickens and red junglefowl demonstrated SK-B1’s closer genetic affinity to existing Thai indigenous and local chicken breeds, suggesting that it may represent a variant within known Thai breeds. In contrast, KU-BM/F, KU-WM/F, and KU-VM/F may be identified as new Phuphan chicken breeds, each categorized as a variety based on plumage morphology. Notably, loci potentially under directional selection, which could harbor adaptive genes linked to plumage color variation, were identified through microsatellite marker analyses. Understanding the genetic architecture and diversity of Phuphan chickens is pivotal for developing targeted breeding strategies. These strategies aim to enhance economic opportunities in Thailand and conserve the genetic diversity within indigenous chicken populations. Future research should explore the adaptive traits and genetic potential to support sustainable agriculture and global food security.

## Figures and Tables

**Table 1:** Genetic diversity among four varieties of Phuphan chickens based on 28 microsatellite loci

Varieties	*N*_a_	*AR*	*N*_ea_	*I*	*H*_o_	*H*_e_	*M* ratio	*PIC*	*F*
SK-B1									
Mean	4.750	4.416	2.991	1.170	0.482	0.615	0.438	0.561	0.196
SE	0.390	1.789	0.230	0.075	0.035	0.029	0.045	0.030	0.048
KU-BM/F									
Mean	4.786	4.636	2.970	1.167	0.652	0.611	0.427	0.556	−0.078
SE	0.379	1.882	0.222	0.074	0.042	0.028	0.062	0.030	0.061
KU-WM/F									
Mean	4.821	4.636	3.114	1.202	0.677	0.625	0.432	0.573	−0.068
SE	0.402	1.994	0.239	0.077	0.044	0.029	0.050	0.031	0.047
KU-VM/F									
Mean	5.214	4.689	3.374	1.267	0.745	0.640	0.401	0.592	−0.168
SE	0.472	2.365	0.304	0.087	0.041	0.031	0.048	0.033	0.039
Total	4.893	4.594	3.112	1.201	0.639	0.623	0.425	0.570	−0.029
SE	0.204	0.124	0.125	0.039	0.022	0.014	0.051	0.031	0.028

*N*_a_, number of alleles; *AR*, allelic richness; *N*_ea_, number of effective alleles; *I*, Shannon’s information index; *H*_o_, observed heterozygosity; *H*_e_, expected heterozygosity; *PIC*, polymorphic information content; *F*, fixation index; SK-B1, Phuphan black 1; KU-BM/F, Phuphan black 2; KU-WM/F, Phuphan white; KU-VM/F, Phuphan color.

## SUPPLEMENTARY MATERIAL

**Supplement 1**. Representative specimens of Phuphan chicken varieties in Thailand.

**Supplement 2**. Genetic diversity of 90 individuals of Phuphan chicken varieties based on 28 microsatellite loci.

**Supplement 3**. Pairwise comparison of genetic differentiation (FST), FST^ENA^ values with ENA correction for null alleles, and R_ST_ values between four Phuphan chicken varieties using FSTAT version 2.9.3 (Goudet, 1995).

**Supplement 4**. Analysis of molecular variance (AMOVA) for Phuphan chicken varieties based on 28 microsatellite loci.

**Supplement 5**. Nei’s genetic distance (D) values between four varieties of Phuphan chicken.

**Supplement 6**. Principal coordinate analysis (PCoA) of four Phuphan chicken varieties based on 28 microsatellite loci.

**Supplement 7**. Discriminant Analysis of Principal Components (DAPC) of four Phuphan chicken varieties.

**Supplement 8**. Population structure of four Phuphan chicken varieties.

**Supplement 9**. Mapping of expected heterozygosity (*H**_e_*) against inbreeding coefficients (*F**_IS_*) along the length of the physical map.

**Supplement 10**. BAYESCAN estimate for the probability of a locus under selection.

**Supplement 11**. Different population structure patterns of Phuphan and other Thai chicken breeds generated by model-based Bayesian clustering algorithms implemented in STRUCTURE.
