# Supplementary material for: Phuphan chicken breeds: classification as varieties or distinct breeds with three derivative groups using microsatellite genotyping
Source: Anim Biosci. 2025 May 19;38(10):2055–66. doi: 10.5713/ab.24.0579 (PMC12415380; doi:10.5713/ab.24.0579)
Supplement: Supplementary file 1 [file ab-24-0579-Supplementary-1.pdf]

Supplement 1. Representative specimens of Phuphan chicken varieties in Thailand

No	Abbreviation/ Code	Breeds	Population	Sex	Locality
1	SK-B1/1	Phuphan Black	Sakon Nakhon (Black)	female	Livestock Royal Development Study Center, Huai Yang
2	SK-B1/2	Phuphan Black	Sakon Nakhon (Black)	male	Livestock Royal Development Study Center, Huai Yang
3	SK-B1/3	Phuphan Black	Sakon Nakhon (Black)	female	Livestock Royal Development Study Center, Huai Yang
4	SK-B1/4	Phuphan Black	Sakon Nakhon (Black)	female	Livestock Royal Development Study Center, Huai Yang
5	SK-B1/5	Phuphan Black	Sakon Nakhon (Black)	male	Livestock Royal Development Study Center, Huai Yang
6	SK-B1/6	Phuphan Black	Sakon Nakhon (Black)	female	Livestock Royal Development Study Center, Huai Yang
7	SK-B1/7	Phuphan Black	Sakon Nakhon (Black)	female	Livestock Royal Development Study Center, Huai Yang
8	SK-B1/8	Phuphan Black	Sakon Nakhon (Black)	female	Livestock Royal Development Study Center, Huai Yang
9	SK-B1/9	Phuphan Black	Sakon Nakhon (Black)	female	Livestock Royal Development Study Center, Huai Yang
10	SK-B1/10	Phuphan Black	Sakon Nakhon (Black)	male	Livestock Royal Development Study Center, Huai Yang
11	SK-B1/11	Phuphan Black	Sakon Nakhon (Black)	female	Livestock Royal Development Study Center, Huai Yang
12	SK-B1/12	Phuphan Black	Sakon Nakhon (Black)	female	Livestock Royal Development Study Center, Huai Yang
13	SK-B1/13	Phuphan Black	Sakon Nakhon (Black)	male	Livestock Royal Development Study Center, Huai Yang
14	SK-B1/14	Phuphan Black	Sakon Nakhon (Black)	male	Livestock Royal Development Study Center, Huai Yang
15	SK-B1/15	Phuphan Black	Sakon Nakhon (Black)	male	Livestock Royal Development Study Center, Huai Yang
16	SK-B1/16	Phuphan Black	Sakon Nakhon (Black)	male	Livestock Royal Development Study Center, Huai Yang
17	SK-B1/17	Phuphan Black	Sakon Nakhon (Black)	male	Livestock Royal Development Study Center, Huai Yang
18	SK-B1/18	Phuphan Black	Sakon Nakhon (Black)	male	Livestock Royal Development Study Center, Huai Yang
19	SK-B1/19	Phuphan Black	Sakon Nakhon (Black)	male	Livestock Royal Development Study Center, Huai Yang
20	SK0-B1/20	Phuphan Black	Sakon Nakhon (Black)	male	Livestock Royal Development Study Center, Huai Yang
21	SK-B1/21	Phuphan Black	Sakon Nakhon (Black)	male	Livestock Royal Development Study Center, Huai Yang
22	SK-B1/22	Phuphan Black	Sakon Nakhon (Black)	male	Livestock Royal Development Study Center, Huai Yang

No	Abbreviation/ Code	Breeds	Population	Sex	Locality
23	SK-B1/23	Phuphan Black	Sakon Nakhon (Black)	male	Livestock Royal Development Study Center, Huai Yang
24	SK-B1/24	Phuphan Black	Sakon Nakhon (Black)	female	Livestock Royal Development Study Center, Huai Yang
25	SK-B1/25	Phuphan Black	Sakon Nakhon (Black)	female	Livestock Royal Development Study Center, Huai Yang
26	SK-B1/26	Phuphan Black	Sakon Nakhon (Black)	female	Livestock Royal Development Study Center, Huai Yang
27	SK-B1/27	Phuphan Black	Sakon Nakhon (Black)	male	Livestock Royal Development Study Center, Huai Yang
28	SK-B1/28	Phuphan Black	Sakon Nakhon (Black)	female	Livestock Royal Development Study Center, Huai Yang
29	SK-B1/29	Phuphan Black	Sakon Nakhon (Black)	female	Livestock Royal Development Study Center, Huai Yang
30	SK-B1/30	Phuphan Black	Sakon Nakhon (Black)	female	Livestock Royal Development Study Center, Huai Yang
31	KU-BM/F	Phuphan Black	Sakon Nakhon (Black)	-	Livestock Royal Development Study Center, Huai Yang
32	KU-BM/F2	Phuphan Black	Sakon Nakhon (Black)	-	Livestock Royal Development Study Center, Huai Yang
33	KU-BM/F3	Phuphan Black	Sakon Nakhon (Black)	-	Livestock Royal Development Study Center, Huai Yang
34	KU-BM/F4	Phuphan Black	Sakon Nakhon (Black)	-	Livestock Royal Development Study Center, Huai Yang
35	KU-BM/F5	Phuphan Black	Sakon Nakhon (Black)	-	Livestock Royal Development Study Center, Huai Yang
36	KU-BM/F6	Phuphan Black	Sakon Nakhon (Black)	-	Livestock Royal Development Study Center, Huai Yang
37	KU-BM/F7	Phuphan Black	Sakon Nakhon (Black)	-	Livestock Royal Development Study Center, Huai Yang
38	KU-BM/F8	Phuphan Black	Sakon Nakhon (Black)	-	Livestock Royal Development Study Center, Huai Yang
39	KU-BM/F9	Phuphan Black	Sakon Nakhon (Black)	-	Livestock Royal Development Study Center, Huai Yang
40	KU-BM/F10	Phuphan Black	Sakon Nakhon (Black)	-	Livestock Royal Development Study Center, Huai Yang
41	KU-BM/F11	Phuphan Black	Sakon Nakhon (Black)	-	Livestock Royal Development Study Center, Huai Yang
42	KU-BM/F12	Phuphan Black	Sakon Nakhon (Black)	-	Livestock Royal Development Study Center, Huai Yang
43	KU-BM/F13	Phuphan Black	Sakon Nakhon (Black)	-	Livestock Royal Development Study Center, Huai Yang
44	KU-BM/F14	Phuphan Black	Sakon Nakhon (Black)	-	Livestock Royal Development Study Center, Huai Yang
45	KU-BM/F15	Phuphan Black	Sakon Nakhon (Black)	-	Livestock Royal Development Study Center, Huai Yang
46	KU-BM/F16	Phuphan Black	Sakon Nakhon (Black)	-	Livestock Royal Development Study Center, Huai Yang

No	Abbreviation/ Code	Breeds	Population	Sex	Locality
47	KU-BM/F17	Phuphan Black	Sakon Nakhon (Black)	-	Livestock Royal Development Study Center, Huai Yang
48	KU-BM/F18	Phuphan Black	Sakon Nakhon (Black)	-	Livestock Royal Development Study Center, Huai Yang
49	KU-BM/F19	Phuphan Black	Sakon Nakhon (Black)	-	Livestock Royal Development Study Center, Huai Yang
50	KU-BM/F20	Phuphan Black	Sakon Nakhon (Black)	-	Livestock Royal Development Study Center, Huai Yang
51	KU-WM/F1	Phuphan Black	Sakon Nakhon (White)	-	Livestock Royal Development Study Center, Huai Yang
52	KU-WM/F2	Phuphan Black	Sakon Nakhon (White)	-	Livestock Royal Development Study Center, Huai Yang
53	KU-WM/F3	Phuphan Black	Sakon Nakhon (White)	-	Livestock Royal Development Study Center, Huai Yang
54	KU-WM/F4	Phuphan Black	Sakon Nakhon (White)	-	Livestock Royal Development Study Center, Huai Yang
55	KU-WM/F5	Phuphan Black	Sakon Nakhon (White)	-	Livestock Royal Development Study Center, Huai Yang
56	KU-WM/F6	Phuphan Black	Sakon Nakhon (White)	-	Livestock Royal Development Study Center, Huai Yang
57	KU-WM/F7	Phuphan Black	Sakon Nakhon (White)	-	Livestock Royal Development Study Center, Huai Yang
58	KU-WM/F8	Phuphan Black	Sakon Nakhon (White)	-	Livestock Royal Development Study Center, Huai Yang
59	KU-WM/F9	Phuphan Black	Sakon Nakhon (White)	-	Livestock Royal Development Study Center, Huai Yang
60	KU-WM/F10	Phuphan Black	Sakon Nakhon (White)	-	Livestock Royal Development Study Center, Huai Yang
61	KU-WM/F11	Phuphan Black	Sakon Nakhon (White)	-	Livestock Royal Development Study Center, Huai Yang
62	KU-WM/F12	Phuphan Black	Sakon Nakhon (White)	-	Livestock Royal Development Study Center, Huai Yang
63	KU-WM/F13	Phuphan Black	Sakon Nakhon (White)	-	Livestock Royal Development Study Center, Huai Yang
64	KU-WM/F14	Phuphan Black	Sakon Nakhon (White)	-	Livestock Royal Development Study Center, Huai Yang
65	KU-WM/F15	Phuphan Black	Sakon Nakhon (White)	-	Livestock Royal Development Study Center, Huai Yang
66	KU-WM/F16	Phuphan Black	Sakon Nakhon (White)	-	Livestock Royal Development Study Center, Huai Yang
67	KU-WM/F17	Phuphan Black	Sakon Nakhon (White)	-	Livestock Royal Development Study Center, Huai Yang
68	KU-WM/F18	Phuphan Black	Sakon Nakhon (White)	-	Livestock Royal Development Study Center, Huai Yang
69	KU-WM/F19	Phuphan Black	Sakon Nakhon (White)	-	Livestock Royal Development Study Center, Huai Yang
70	KU-WM/F20	Phuphan Black	Sakon Nakhon (White)	-	Livestock Royal Development Study Center, Huai Yang

No	Abbreviation/ Code	Breeds	Population	Sex	Locality
71	KU-VM/F1	Phuphan Black	Sakon Nakhon (Color)	-	Livestock Royal Development Study Center, Huai Yang
72	KU-VM/F2	Phuphan Black	Sakon Nakhon (Color)	-	Livestock Royal Development Study Center, Huai Yang
73	KU-VM/F3	Phuphan Black	Sakon Nakhon (Color)	-	Livestock Royal Development Study Center, Huai Yang
74	KU-VM/F4	Phuphan Black	Sakon Nakhon (Color)	-	Livestock Royal Development Study Center, Huai Yang
75	KU-VM/F5	Phuphan Black	Sakon Nakhon (Color)	-	Livestock Royal Development Study Center, Huai Yang
76	KU-VM/F6	Phuphan Black	Sakon Nakhon (Color)	-	Livestock Royal Development Study Center, Huai Yang
77	KU-VM/F7	Phuphan Black	Sakon Nakhon (Color)	-	Livestock Royal Development Study Center, Huai Yang
78	KU-VM/F8	Phuphan Black	Sakon Nakhon (Color)	-	Livestock Royal Development Study Center, Huai Yang
79	KU-VM/F9	Phuphan Black	Sakon Nakhon (Color)	-	Livestock Royal Development Study Center, Huai Yang
80	KU-VM/F10	Phuphan Black	Sakon Nakhon (Color)	-	Livestock Royal Development Study Center, Huai Yang
81	KU-VM/F11	Phuphan Black	Sakon Nakhon (Color)	-	Livestock Royal Development Study Center, Huai Yang
82	KU-VM/F12	Phuphan Black	Sakon Nakhon (Color)	-	Livestock Royal Development Study Center, Huai Yang
83	KU-VM/F13	Phuphan Black	Sakon Nakhon (Color)	-	Livestock Royal Development Study Center, Huai Yang
84	KU-VM/F14	Phuphan Black	Sakon Nakhon (Color)	-	Livestock Royal Development Study Center, Huai Yang
85	KU-VM/F15	Phuphan Black	Sakon Nakhon (Color)	-	Livestock Royal Development Study Center, Huai Yang
86	KU-VM/F16	Phuphan Black	Sakon Nakhon (Color)	-	Livestock Royal Development Study Center, Huai Yang
87	KU-VM/F17	Phuphan Black	Sakon Nakhon (Color)	-	Livestock Royal Development Study Center, Huai Yang
88	KU-VM/F18	Phuphan Black	Sakon Nakhon (Color)	-	Livestock Royal Development Study Center, Huai Yang
89	KU-VM/F19	Phuphan Black	Sakon Nakhon (Color)	-	Livestock Royal Development Study Center, Huai Yang
90	KU-VM/F20	Phuphan Black	Sakon Nakhon (Color)	-	Livestock Royal Development Study Center, Huai Yang
