# Supplementary material for: Phuphan chicken breeds: classification as varieties or distinct breeds with three derivative groups using microsatellite genotyping
Source: Anim Biosci. 2025 May 19;38(10):2055–66. doi: 10.5713/ab.24.0579 (PMC12415380; doi:10.5713/ab.24.0579)
Supplement: Supplementary file 2 [file ab-24-0579-Supplementary-2.pdf]

Supplement 2. Genetic diversity of 90 individuals of Phuphan chicken varieties based on 28 microsatellite loci

Varieties	Locus	N	N_a[1]	*AR*[2]	N_{ea}[3]	*I*[4]	H_o[5]	H_e[6]	*M* ratio[7]	*PIC*[8]	*F*[9]	*HWE*[10]
	MCW0111	30	5.000	4.816	4.100	1.468	0.800	0.756	0.357	0.714	–0.058	ns
	LEI0234	30	9.000	8.302	5.158	1.877	0.867	0.806	0.063	0.783	–0.075	ns
	MCW0206	30	4.000	3.957	2.568	1.095	0.567	0.611	0.182	0.543	0.072	ns
	MCW0016	30	5.000	4.383	2.975	1.223	0.300	0.664	0.417	0.605	0.548	***
	MCW0103	30	2.000	2.000	1.923	0.673	0.667	0.480	0.500	0.365	–0.389	*
	MCW0014	30	4.000	4.000	3.666	1.340	0.267	0.727	0.167	0.677	0.633	***
	ADL0268	29	4.000	3.974	2.346	1.070	0.517	0.574	0.500	0.527	0.098	ns
	MCW0034	28	8.000	7.827	6.938	1.992	0.357	0.856	0.500	0.839	0.583	***
	LEI0166	27	3.000	2.867	1.965	0.780	0.444	0.491	0.300	0.402	0.095	Ns
	MCW0037	28	3.000	3.000	2.626	1.028	0.357	0.619	0.750	0.547	0.423	*
	MCW0295	30	8.000	6.624	4.839	1.695	0.733	0.793	0.571	0.761	0.076	***
	LEI0094	28	8.000	7.249	2.987	1.483	0.429	0.665	0.235	0.636	0.356	***
	MCW0216	28	7.000	5.766	3.207	1.387	0.464	0.688	0.292	0.642	0.325	ns
SK-B1[11]	MCW0222	30	3.000	3.000	2.476	1.000	0.567	0.596	0.500	0.530	0.049	ns
	MCW0098	28	2.000	1.850	1.074	0.154	0.071	0.069	1.000	0.067	–0.037	ns
	MCW0078	30	2.000	2.000	1.642	0.580	0.467	0.391	0.500	0.315	–0.193	ns
	MCW0081	30	4.000	3.923	2.740	1.136	0.667	0.635	0.200	0.569	–0.050	ns
	MCW0183	29	3.000	2.999	1.842	0.808	0.379	0.457	0.188	0.413	0.170	ns
	MCW0067	30	5.000	4.816	3.789	1.425	0.833	0.736	0.625	0.691	–0.132	ns
	MCW0248	29	7.000	5.749	3.168	1.397	0.448	0.684	0.583	0.642	0.345	**
	LEI0192	27	7.000	6.106	2.531	1.292	0.296	0.605	0.194	0.573	0.510	***
	ADL0112	28	4.000	3.978	2.243	1.041	0.429	0.554	0.667	0.510	0.227	**
	MCW0165	28	3.000	3.000	2.649	1.035	0.357	0.622	0.750	0.551	0.426	**
	ADL0278	27	3.000	2.630	2.017	0.758	0.296	0.504	0.750	0.394	0.412	ns
	MCW0104	29	4.000	3.833	2.670	1.115	0.414	0.625	0.154	0.563	0.338	***
	MCW0123	30	4.000	3.565	2.479	1.028	0.467	0.597	0.667	0.520	0.218	***
	MCW0330	29	5.000	4.558	2.808	1.206	0.517	0.644	0.156	0.583	0.197	ns
	MCW0069	29	7.000	6.866	4.313	1.671	0.517	0.768	0.500	0.736	0.327	***
	Mean	28.964	4.750	4.416	2.991	1.170	0.482	0.615	0.438	0.561	0.196	
	SE	0.202	0.390	1.789	0.230	0.075	0.035	0.029	0.045	0.030	0.048	
	MCW0111	20	5.000	4.979	2.807	1.259	0.450	0.644	0.875	0.597	0.301	*
	LEI0234	20	7.000	6.831	5.556	1.786	0.650	0.820	0.046	0.795	0.207	ns
	MCW0206	20	4.000	3.700	2.116	0.870	0.400	0.528	0.409	0.433	0.242	ns
	MCW0016	20	9.000	8.512	5.442	1.863	0.950	0.816	0.265	0.792	–0.164	ns

Varieties	Locus	N	N_a[1]	AR[2]	N_{ea}[3]	I[4]	H_o[5]	H_e[6]	M ratio[7]	PIC[8]	F[9]	HWE[10]
KU-BM/F[12]	MCW0103	20	3.000	3.000	2.597	1.010	0.700	0.615	1.000	0.534	–0.138	*
	MCW0014	20	6.000	5.961	4.233	1.572	0.650	0.764	0.273	0.727	0.149	*
	ADL0268	20	4.000	3.850	2.909	1.145	0.800	0.656	0.833	0.587	–0.219	*
	MCW0034	20	5.000	4.831	2.985	1.256	0.800	0.665	0.278	0.610	–0.203	ns
	LEI0166	20	5.000	4.662	1.372	0.622	0.300	0.271	0.167	0.262	–0.106	ns
	MCW0037	20	2.000	2.000	1.980	0.688	0.700	0.495	1.000	0.372	–0.414	ns
	MCW0295	20	5.000	4.998	4.420	1.535	1.000	0.774	0.429	0.736	–0.292	ns
	LEI0094	17	6.000	6.000	4.516	1.607	0.882	0.779	0.250	0.744	–0.133	ns
	MCW0216	20	9.000	8.529	4.678	1.808	0.800	0.786	0.100	0.762	–0.017	ns
	MCW0222	20	4.000	3.700	1.732	0.753	0.300	0.423	0.667	0.368	0.290	***
	MCW0098	20	2.000	2.000	1.536	0.533	0.350	0.349	1.000	0.288	–0.004	ns
	MCW0078	20	2.000	2.000	1.995	0.692	0.950	0.499	1.000	0.374	–0.905	***
	MCW0081	20	6.000	5.698	3.089	1.347	0.600	0.676	0.179	0.628	0.113	ns
	MCW0183	19	5.000	5.000	3.861	1.468	0.421	0.741	0.250	0.700	0.432	***
	MCW0067	20	5.000	4.831	2.778	1.221	0.900	0.640	0.375	0.590	–0.406	ns
	MCW0248	20	3.000	3.000	2.492	0.980	0.500	0.599	0.375	0.514	0.165	ns
	LEI0192	20	8.000	7.529	2.730	1.429	0.350	0.634	0.055	0.609	0.448	**
	ADL0112	20	7.000	6.827	3.922	1.590	0.800	0.745	0.438	0.708	–0.074	**
	MCW0165	20	3.000	3.000	2.572	1.006	0.900	0.611	0.750	0.531	–0.472	*
	ADL0278	20	3.000	3.000	2.046	0.876	0.500	0.511	0.300	0.454	0.022	ns
	MCW0104	20	6.000	5.400	1.533	0.778	0.350	0.348	0.050	0.331	–0.007	***
	MCW0123	20	2.000	2.000	1.956	0.682	0.850	0.489	0.067	0.369	–0.739	***
	MCW0330	20	4.000	4.000	2.694	1.178	0.600	0.629	0.125	0.584	0.046	ns
	MCW0069	20	4.000	3.981	2.606	1.109	0.800	0.616	0.400	0.553	–0.298	ns
	Mean	19.857	4.786	4.636	2.970	1.167	0.652	0.611	0.427	0.556	–0.078	
	SE	0.112	0.379	1.882	0.222	0.074	0.042	0.028	0.062	0.030	0.061	
	MCW0111	20	4.000	4.000	3.865	1.369	0.850	0.741	0.667	0.694	–0.147	ns
	LEI0234	20	8.000	7.531	4.082	1.650	0.600	0.755	0.052	0.723	0.205	**
	MCW0206	20	3.000	2.981	2.111	0.836	0.500	0.526	0.300	0.431	0.050	ns
	MCW0016	20	7.000	6.696	3.687	1.543	0.900	0.729	0.583	0.692	–0.235	***
	MCW0103	19	3.000	2.895	1.667	0.662	0.474	0.400	0.500	0.339	–0.183	ns
	MCW0014	20	6.000	5.698	3.738	1.448	0.900	0.733	0.261	0.686	–0.229	ns
	ADL0268	20	5.000	4.848	3.419	1.344	0.900	0.708	0.714	0.656	–0.272	ns
	MCW0034	19	3.000	2.991	1.615	0.673	0.263	0.381	0.250	0.338	0.309	***
	LEI0166	20	3.000	2.850	1.354	0.490	0.200	0.261	0.300	0.238	0.234	ns
	MCW0037	20	2.000	2.000	1.663	0.588	0.350	0.399	1.000	0.319	0.122	ns
	MCW0295	20	5.000	4.831	3.433	1.326	0.750	0.709	0.357	0.653	–0.058	ns

Varieties	Locus	N	N_a[1]	*AR*[2]	N_{ea}[3]	*I*[4]	H_o[5]	H_e[6]	*M* ratio[7]	*PIC*[8]	*F*[9]	*HWE*[10]
	LEI0094	20	12.000	11.342	6.838	2.169	0.900	0.854	0.333	0.839	–0.054	*
	MCW0216	20	6.000	5.698	2.581	1.249	0.450	0.613	0.150	0.573	0.265	ns
KU-WM/F[13]	MCW0222	20	4.000	3.998	2.589	1.130	0.550	0.614	0.667	0.560	0.104	ns
	MCW0098	20	2.000	2.000	1.600	0.562	0.400	0.375	1.000	0.305	–0.067	ns
	MCW0078	20	3.000	2.998	2.266	0.899	0.950	0.559	0.500	0.466	–0.700	***
	MCW0081	20	5.000	4.981	3.571	1.399	0.500	0.720	0.208	0.672	0.306	ns
	MCW0183	19	4.000	3.895	1.957	0.914	0.316	0.489	0.250	0.448	0.354	ns
	MCW0067	19	5.000	4.886	3.327	1.314	0.842	0.699	0.625	0.643	–0.204	ns
	MCW0248	20	4.000	3.848	2.260	0.970	0.700	0.558	0.667	0.482	–0.256	ns
	LEI0192	20	8.000	7.828	5.714	1.879	0.800	0.825	0.063	0.803	0.030	ns
	ADL0112	20	5.000	4.998	4.444	1.537	1.000	0.775	0.625	0.738	–0.290	ns
	MCW0165	20	3.000	3.000	2.417	0.966	0.850	0.586	0.750	0.506	–0.450	*
	ADL0278	20	4.000	3.850	2.614	1.074	0.750	0.618	0.400	0.542	–0.215	ns
	MCW0104	20	6.000	5.831	3.404	1.438	0.700	0.706	0.150	0.665	0.009	***
	MCW0123	20	5.000	4.981	4.061	1.480	0.850	0.754	0.156	0.713	–0.128	**
	MCW0330	20	4.000	3.998	3.200	1.249	0.850	0.688	0.125	0.631	–0.236	*
	MCW0069	20	6.000	5.848	3.704	1.501	0.850	0.730	0.429	0.693	–0.164	ns
	Mean	19.857	4.821	4.636	3.114	1.202	0.677	0.625	0.432	0.573	–0.068	
	SE	0.067	0.402	1.994	0.239	0.077	0.044	0.029	0.050	0.031	0.047	
	MCW0111	20	5.000	4.850	3.509	1.381	0.800	0.715	0.357	0.670	–0.119	ns
	LEI0234	20	11.000	10.525	7.143	2.151	1.000	0.860	0.072	0.846	–0.163	ns
	MCW0206	20	5.000	4.848	2.963	1.248	0.700	0.663	0.227	0.601	–0.057	ns
	MCW0016	20	8.000	7.531	4.167	1.667	1.000	0.760	0.444	0.730	–0.316	**
	MCW0103	19	4.000	3.895	2.645	1.084	1.000	0.622	0.667	0.548	–0.608	**
	MCW0014	20	6.000	5.700	4.188	1.523	0.850	0.761	0.250	0.721	–0.117	***
	ADL0268	20	3.000	3.000	2.477	1.000	0.900	0.596	0.750	0.530	–0.509	**
	MCW0034	20	4.000	3.850	2.210	0.996	0.500	0.548	0.133	0.497	0.087	ns
	LEI0166	19	2.000	2.000	1.951	0.681	0.526	0.488	0.333	0.369	–0.080	ns
	MCW0037	20	3.000	2.850	1.559	0.612	0.450	0.359	0.750	0.310	–0.254	ns
	MCW0295	20	8.000	7.660	4.124	1.672	0.850	0.758	0.444	0.727	–0.122	ns
	LEI0094	20	11.000	10.528	8.081	2.208	0.750	0.876	0.324	0.864	0.144	**
	MCW0216	20	6.000	5.812	2.996	1.345	0.550	0.666	0.158	0.622	0.174	*
KU-VM/F[14]	MCW0222	20	3.000	3.000	2.247	0.938	0.600	0.555	0.500	0.491	–0.081	ns
	MCW0098	20	2.000	1.998	1.161	0.266	0.150	0.139	1.000	0.129	–0.081	ns
	MCW0078	20	4.000	4.000	3.200	1.255	1.000	0.688	0.667	0.630	–0.455	**
	MCW0081	20	5.000	4.981	3.404	1.389	0.850	0.706	0.208	0.664	–0.204	ns
	MCW0183	19	4.000	3.895	1.748	0.825	0.421	0.428	0.250	0.396	0.016	ns

Varieties	Locus	N	N_a[1]	*AR*[2]	N_{ea}[3]	*I*[4]	H_o[5]	H_e[6]	*M* ratio[7]	*PIC*[8]	*F*[9]	*HWE*[10]
	MCW0067	19	4.000	3.895	2.674	1.089	1.000	0.626	0.667	0.551	–0.597	*
	MCW0248	20	2.000	2.000	1.882	0.662	0.650	0.469	0.500	0.359	–0.387	ns
	LEI0192	19	9.000	8.667	4.198	1.757	0.684	0.762	0.071	0.738	0.102	*
	ADL0112	20	5.000	4.981	3.721	1.427	0.950	0.731	0.625	0.686	–0.299	***
	MCW0165	20	3.000	3.000	2.589	1.017	0.700	0.614	0.750	0.539	–0.141	ns
	ADL0278	19	4.000	4.000	3.099	1.227	0.737	0.677	0.500	0.617	–0.088	ns
	MCW0104	20	5.000	4.829	2.020	1.007	0.650	0.505	0.132	0.469	–0.287	ns
	MCW0123	20	7.000	6.831	5.096	1.743	0.950	0.804	0.206	0.776	–0.182	ns
	MCW0330	19	5.000	4.991	3.703	1.428	0.684	0.730	0.156	0.686	0.063	ns
	MCW0069	20	8.000	7.811	5.714	1.869	0.950	0.825	0.075	0.802	–0.152	ns
	Mean	19.750	5.214	4.689	3.374	1.267	0.745	0.640	0.401	0.592	–0.168	
	SE	0.083	0.472	2.365	0.304	0.087	0.041	0.031	0.048	0.033	0.039	

[1]Number of alleles (N_a); [2]allelic richness (*AR*); [3]number of effective alleles (N_{ea}); [4]Shannon's information index (*I*); [5]observed heterozygosity (H_o); [6]expected heterozygosity (H_e); [7]*M* ratio; [8]polymorphic information content (*PIC*); [9]fixation index (*F*), [10]*HWE* (Hardy-Weinberg Equilibrium); ns, not significant; *, $p<0.05$; **, $p<0.01$; ***, $p<0.001$, [11]Phuphan black 1 (SK-B1), [12]Phuphan black 2 (KU-BM/F), [13]Phuphan white (KU-WM/F), [14]Phuphan color (KU-VM/F)
