# Supplementary material for: Phuphan chicken breeds: classification as varieties or distinct breeds with three derivative groups using microsatellite genotyping
Source: Anim Biosci. 2025 May 19;38(10):2055–66. doi: 10.5713/ab.24.0579 (PMC12415380; doi:10.5713/ab.24.0579)
Supplement: Supplementary file 3 [file ab-24-0579-Supplementary-3.pdf]

Supplement 3. Pairwise comparison of genetic differentiation (F_{ST}), F_{ST}^{ENA} values with ENA correction for null alleles, and R_{ST} values between four Phuphan chicken varieties using FSTAT version 2.9.3 (Goudet, 1995). The numbers indicate *p*-values with 110 permutations

Combination	F_{ST}	F_{ST}^{ENA}	R_{ST}
SK-B1 x KU-BM/F	0.223*	0.218	0.185
SK-B1 x KU-WM/F	0.199*	0.193	0.194
SK-B1 x KU-VM/F	0.161*	0.161	0.175
KU-BM/F x KU-WM/F	0.066*	0.061	0.063
KU-BM/F x KU-VM/F	0.077*	0.075	0.075
KU-WM/F x KU-VM/F	0.046*	0.045	0.059

*p-value < 0.05. SK-B1 = Phuphan black 1; KU-BM/F = Phuphan black 2; KU-WM/F = Phuphan white; KU-VM/F = Phuphan color
