# Supplementary material for: Phuphan chicken breeds: classification as varieties or distinct breeds with three derivative groups using microsatellite genotyping
Source: Anim Biosci. 2025 May 19;38(10):2055–66. doi: 10.5713/ab.24.0579 (PMC12415380; doi:10.5713/ab.24.0579)
Supplement: Supplementary file 4 [file ab-24-0579-Supplementary-4.pdf]

Supplement 4. Analysis of molecular variance (AMOVA) for Phuphan Chicken varieties based on 28 microsatellite loci

Source of variation	df	Sum of squares	Variance components	Percentage of variation
among populations	3	224.575	1.468	14%
among individual	86	826.625	0.514	5%
within individual	90	772.500	8.583	81%
Total	179	1823.700	10.566	100%

df = degree of freedom
