# Supplementary material for: Phuphan chicken breeds: classification as varieties or distinct breeds with three derivative groups using microsatellite genotyping
Source: Anim Biosci. 2025 May 19;38(10):2055–66. doi: 10.5713/ab.24.0579 (PMC12415380; doi:10.5713/ab.24.0579)
Supplement: Supplementary file 5 [file ab-24-0579-Supplementary-5.pdf]

Supplement 5. Nei's genetic distance (*D*) values between four varieties of Phuphan chicken.

Nei's *D*	SK-B1	KU-BM/F	KU-WM/F	KU-VM/F
SK-B1	0.000			
KU-BM/F	0.667	0.000		
KU-WM/F	0.570	0.128	0.000	
KU-VM/F	0.425	0.157	0.088	0.000

SK-B1 = Phuphan black 1; KU-BM/F = Phuphan black 2; KU-WM/F = Phuphan white; KU-VM/F = Phuphan color
