# Supplementary material for: Phuphan chicken breeds: classification as varieties or distinct breeds with three derivative groups using microsatellite genotyping
Source: Anim Biosci. 2025 May 19;38(10):2055–66. doi: 10.5713/ab.24.0579 (PMC12415380; doi:10.5713/ab.24.0579)
Supplement: Supplementary file 6 [file ab-24-0579-Supplementary-6.pdf]

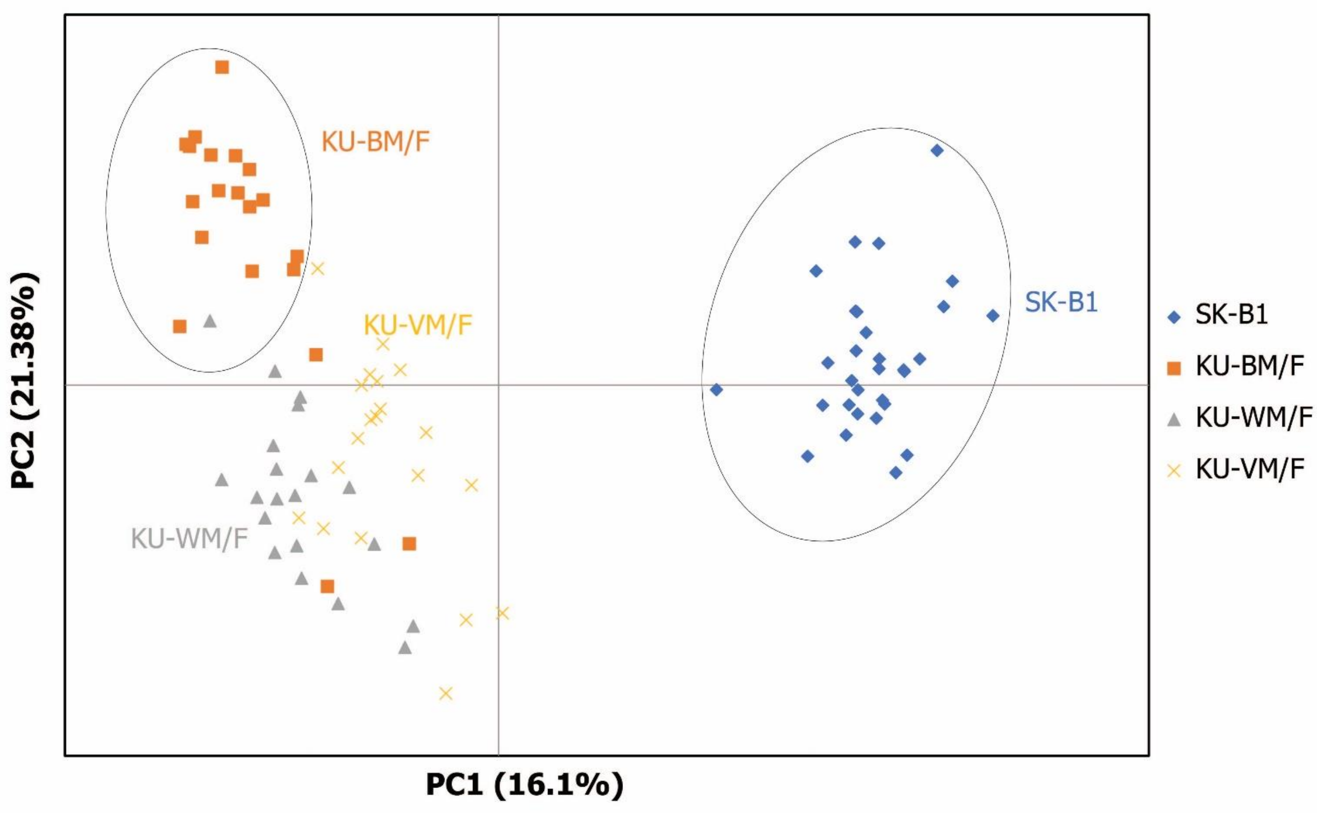

Supplement 6. Principal coordinate analysis (PCoA) of four Phuphan chicken varieties based on 28 microsatellite loci.
