# Supplementary material for: Phuphan chicken breeds: classification as varieties or distinct breeds with three derivative groups using microsatellite genotyping
Source: Anim Biosci. 2025 May 19;38(10):2055–66. doi: 10.5713/ab.24.0579 (PMC12415380; doi:10.5713/ab.24.0579)
Supplement: Supplementary file 7 [file ab-24-0579-Supplementary-7.pdf]

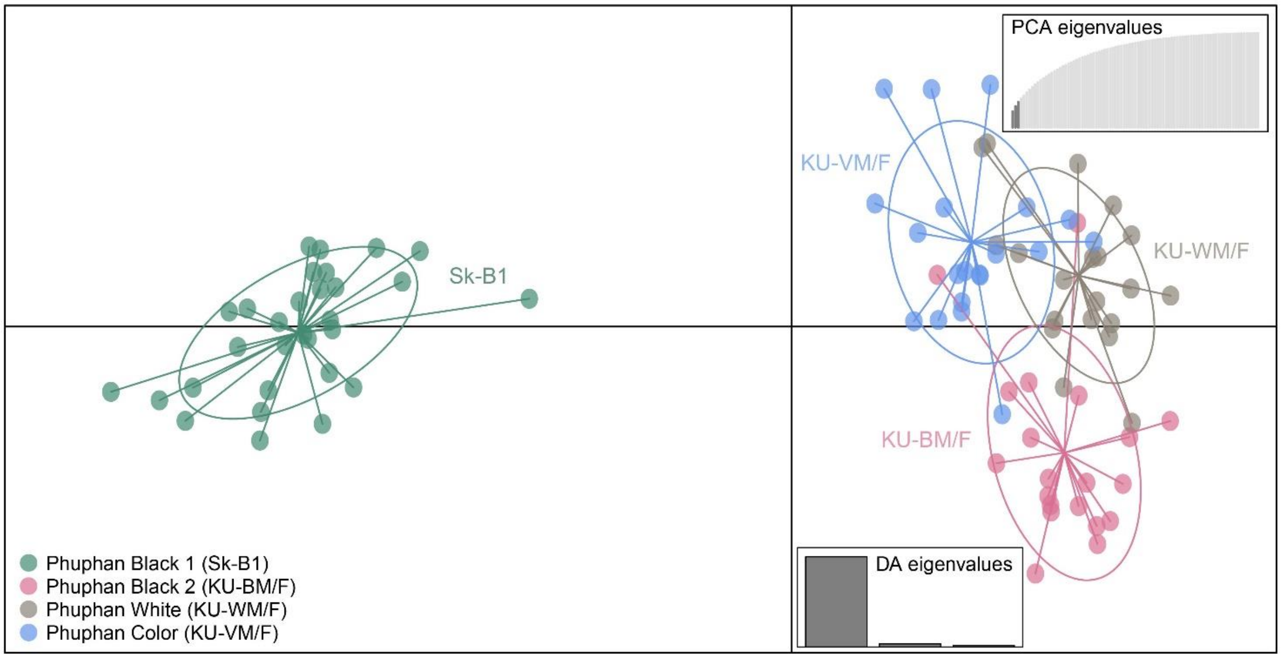

Supplement 7. Discriminant Analysis of Principal Components (DAPC) of four Phuphan chicken varieties. Scatter plots based on DAPC output for assigned genetic clusters are indicated by different colors. Dots represent different individuals.
