# Supplementary material for: Phuphan chicken breeds: classification as varieties or distinct breeds with three derivative groups using microsatellite genotyping
Source: Anim Biosci. 2025 May 19;38(10):2055–66. doi: 10.5713/ab.24.0579 (PMC12415380; doi:10.5713/ab.24.0579)
Supplement: Supplementary file 8 [file ab-24-0579-Supplementary-8.pdf]

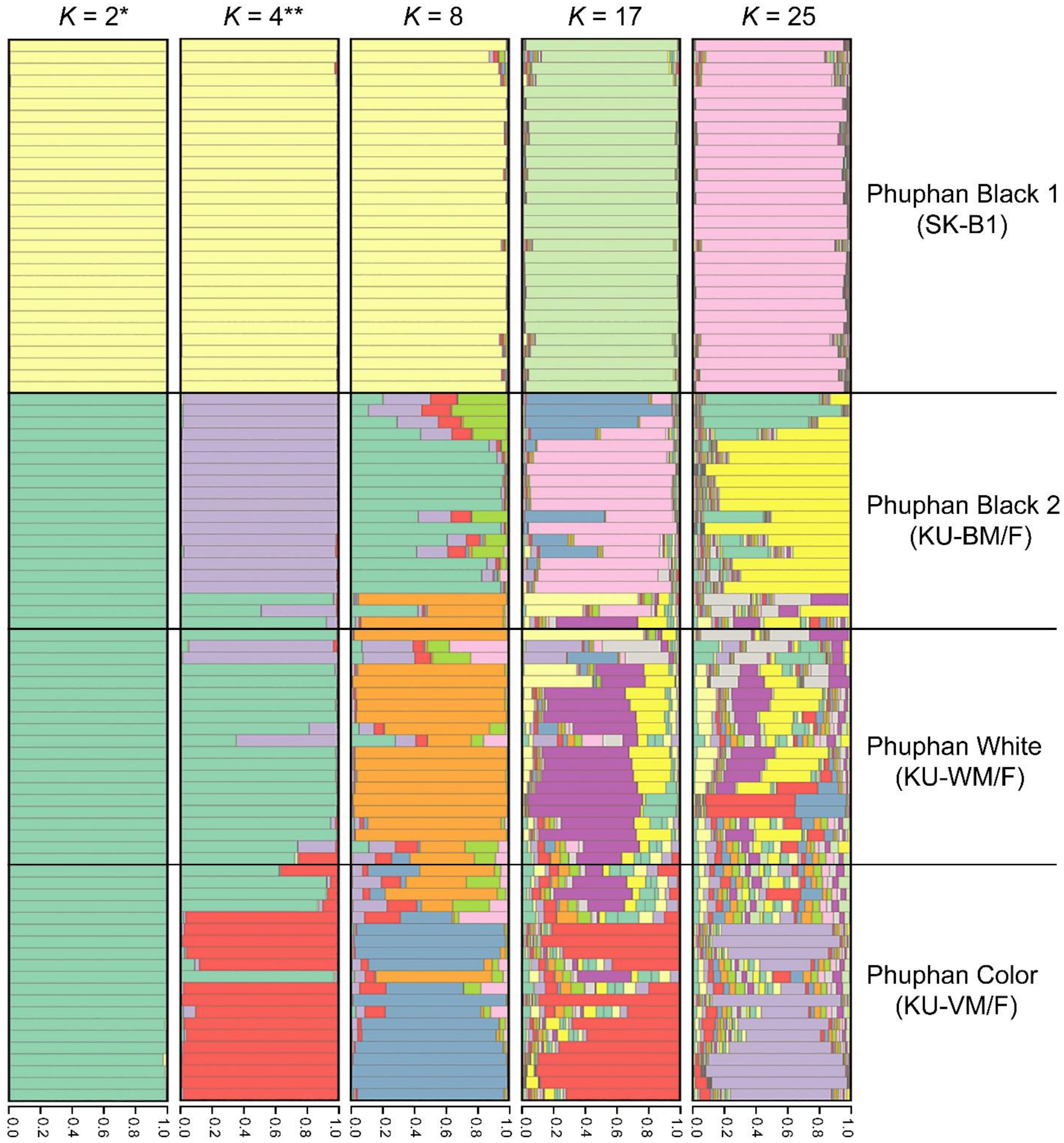

Supplement 8. Population structure of four Phuphan chicken varieties. The *x*-axis represents the proportion of membership (posterior probability) in each genetic cluster, while each horizontal bar on the *y*-axis represents an individual. All individuals from the four varieties are superimposed on the plot. Black vertical lines indicate the boundaries. The highest posterior probability, denoted by *, was determined based on Evanno's Δ*K*, and the highest ln P(*K*) is represented by **.
