# Supplementary material for: Phuphan chicken breeds: classification as varieties or distinct breeds with three derivative groups using microsatellite genotyping
Source: Anim Biosci. 2025 May 19;38(10):2055–66. doi: 10.5713/ab.24.0579 (PMC12415380; doi:10.5713/ab.24.0579)
Supplement: Supplementary file 9 [file ab-24-0579-Supplementary-9.pdf]

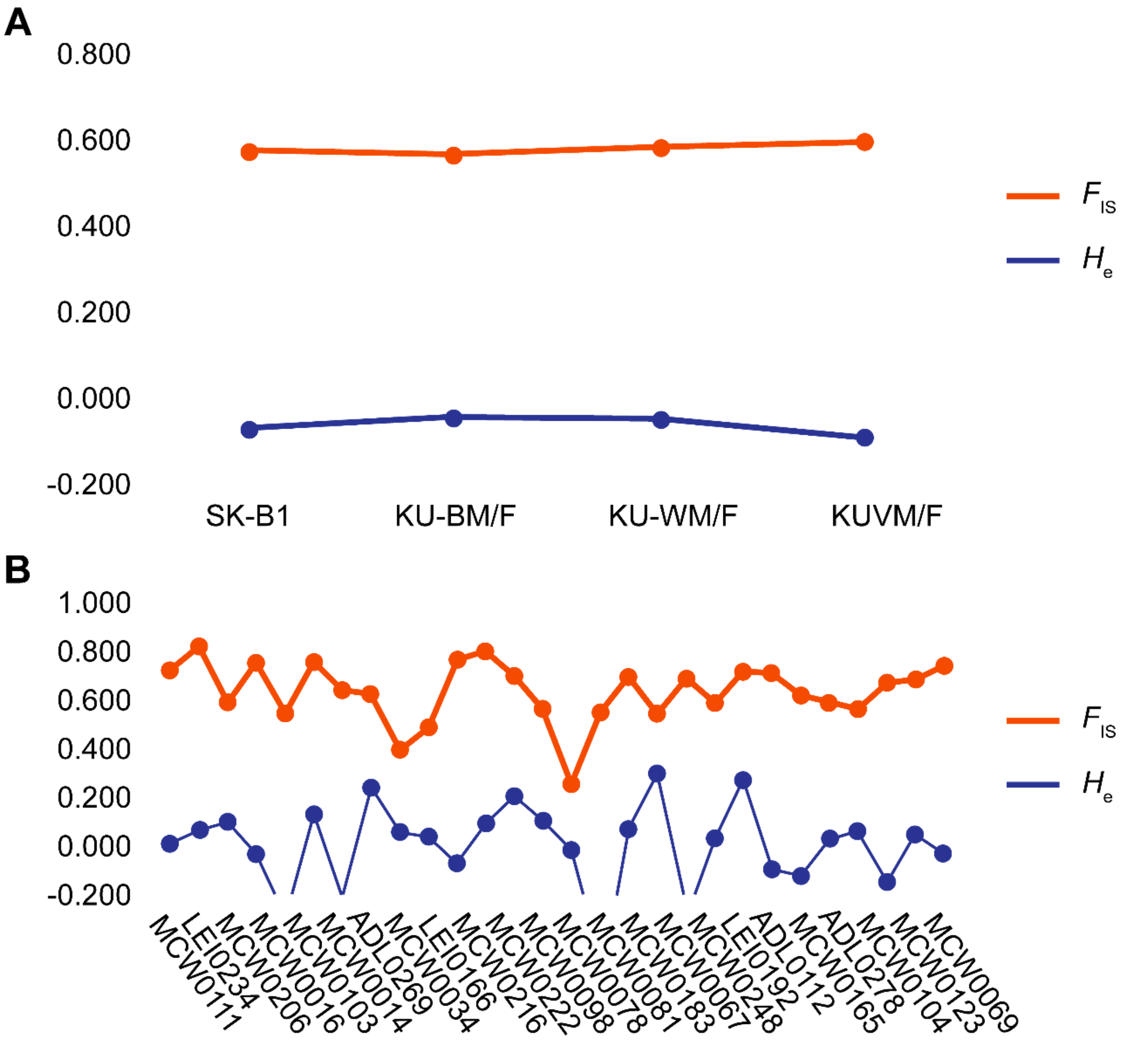

Supplement 9. Mapping of expected heterozygosity (H_e) against inbreeding coefficients (F_{IS}) along the length of the physical map. (A) Four Phuphan chicken varieties (B) Microsatellite loci for Phuphan chicken.
