# Supplementary material for: Phuphan chicken breeds: classification as varieties or distinct breeds with three derivative groups using microsatellite genotyping
Source: Anim Biosci. 2025 May 19;38(10):2055–66. doi: 10.5713/ab.24.0579 (PMC12415380; doi:10.5713/ab.24.0579)
Supplement: Supplementary file 10 [file ab-24-0579-Supplementary-10.pdf]

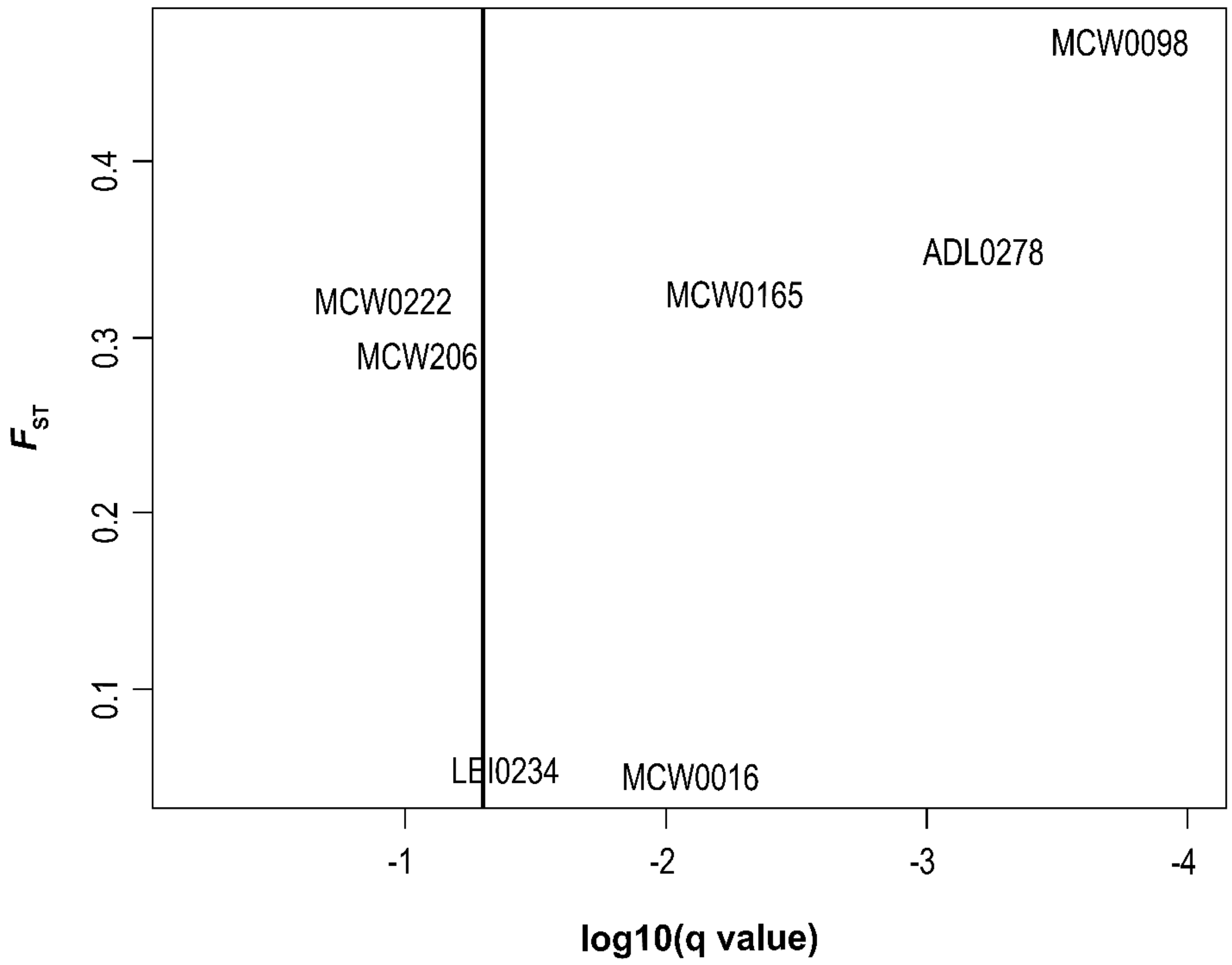

Supplement 10. BAYESCAN estimate for the probability of a locus under selection. Results of F_{ST} values for each locus are plotted against their log-transformed Bayes factor.
