# Supplementary material for: Phuphan chicken breeds: classification as varieties or distinct breeds with three derivative groups using microsatellite genotyping
Source: Anim Biosci. 2025 May 19;38(10):2055–66. doi: 10.5713/ab.24.0579 (PMC12415380; doi:10.5713/ab.24.0579)
Supplement: Supplementary file 11 [file ab-24-0579-Supplementary-11.pdf]

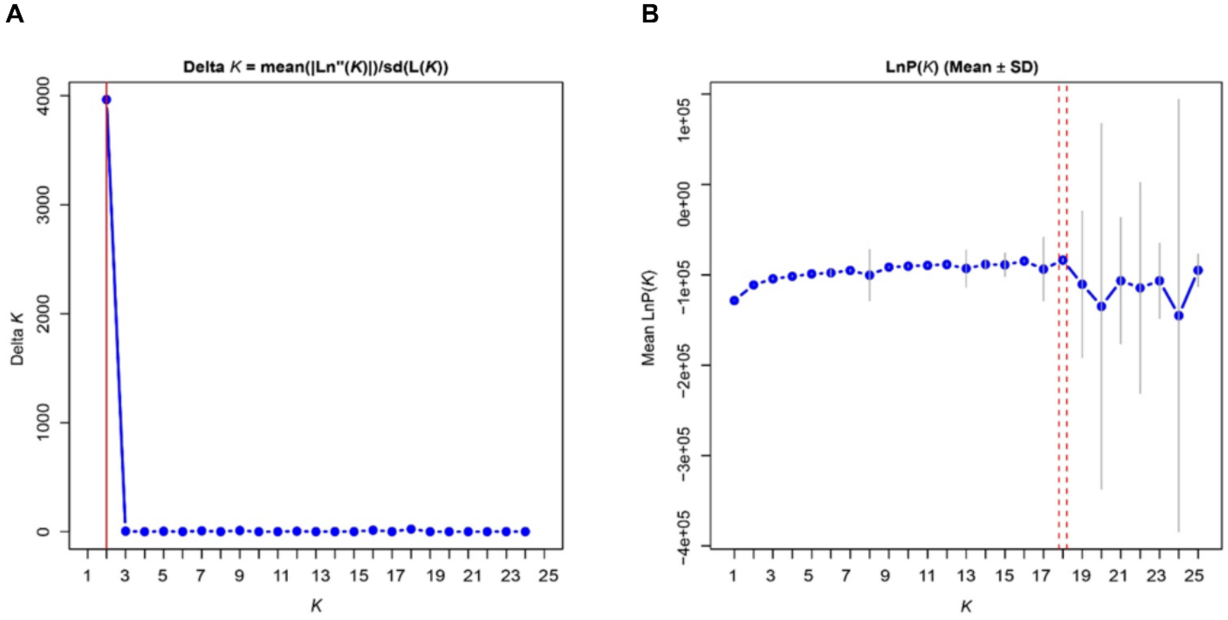

Supplement 11. Different population structure patterns of Phuphan and other Thai chicken breeds generated by model-based Bayesian clustering algorithms implemented in STRUCTURE. (A) Plot of Evanno's Δ*K* and (B) Plot of LnP(*K*).
